# Flexible Boron-Doped Diamond (BDD) Electrodes for Plant Monitoring

**DOI:** 10.3390/s17071638

**Published:** 2017-07-15

**Authors:** Shoko Tago, Tsuyoshi Ochiai, Seitaro Suzuki, Mio Hayashi, Takeshi Kondo, Akira Fujishima

**Affiliations:** 1 Photocatalyst Group, Research and Development Department, Local Independent Administrative Agency Kanagawa Institute of industrial Science and TEChnology (KISTEC), 407 East Wing, Innovation Center Building, KSP, 3-2-1 Sakado, Takatsu-ku, Kawasaki, Kanagawa 213-0012, Japan; pg-tago@newkast.or.jp (S.T.); c1145247@cstu.nit.ac.jp (S.S.); pg-hayashi@newkast.or.jp (M.H.); fujishima_akira@admin.tus.ac.jp (A.F.); 2 Materials Analysis Group, Kawasaki Technical Support Department, KISTEC, Ground Floor East Wing, Innovation Center Building, KSP, 3-2-1 Sakado, Takatsu-ku, Kawasaki, Kanagawa 213-0012, Japan; 3 Photocatalysis International Research Centre, Tokyo University of Science, 2641 Yamazaki, Noda, Chiba 278-8510, Japan; t-kondo@rs.noda.tus.ac.jp; 4 Nippon Institute of Technology, 4-1 Gakuendai, Miyashiro-machi, Minamisaitama-gun, Saitama 345-8501, Japan

**Keywords:** boron-doped diamond (BDD) electrode, bioelectric potentials, plant monitoring, flexible sensor electrode

## Abstract

Detecting the bio-potential changes of plants would be useful for monitoring their growth and health in the field. A sensitive plant monitoring system with flexible boron-doped diamond (BDD) electrodes prepared from BDD powder and resin (Nafion or Vylon-KE1830) was investigated. The properties of the electrodes were compared with those of small BDD plate-type electrodes by monitoring the bioelectric potentials of potted *Aloe* and hybrid species in the genus *Opuntia*. While flexible BDD electrodes have wide potential windows, their cyclic voltammograms are different from those of the BDD plate. Further, the potential gap between a pair of electrodes attached to the plants changes as the plants are stimulated artificially with a finger touch, suggesting that the bioelectric potentials in the plant also changed, manifesting as changes in the potential gap between the electrodes. The BDD electrodes were assessed for their response reproducibility to a finger stimulus for 30 days. It was concluded that the plant monitoring system worked well with flexible BDD electrodes. Further, the electrodes were stable, and as reliable as the BDD plate electrodes in this study. Thus, a flexible and inexpensive BDD electrode system was successfully fabricated for monitoring the bioelectric potential changes in plants.

## 1. Introduction

Plants are capable of detecting environmental factors such as atmospheric temperature, humidity, and light intensity. It has become obvious that the bioelectric potential of plants is influenced by environmental factors, and changes in the environmental factors can be detected by monitoring changes in the bioelectric potential [1,2,3,4,5,6]. Previously, we reported a highly sensitive measurement system involving boron-doped diamond (BDD) electrodes for plant monitoring [7,8]. However, although the BDD electrodes seemed to be promising for plant monitoring [9,10], further research is needed for the development of inexpensive, stable systems that can be coupled with a home PC to monitor plants in the field.

Fierro et al., have reported BDD microelectrodes for monitoring pH changes in vivo [11] and assessing cancerous tumors [12]. Reward-induced burst firing of dopaminergic neurons in the monkey brain [13] has also been detected with BDD microelectrodes by Yoshimi et al. Although the properties of BDD microelectrodes are promising, the electrodes are still expensive and susceptible to breakage while being set up on plants.

Therefore, in the current study, the application of flexible and low cost BDD electrodes prepared using BDD powder and resins is described for long-term plant monitoring. We have already reported the use of flexible BDD electrodes prepared using BDD powder and Nafion for disinfecting root canals in dental treatment [14]. The BDD/Nafion ink was thought to be suitable for the fabrication of flexible BDD films. Further, Kondo et al., reported the use of BDD/Vylon ink for preparing screen-printed diamond electrodes based on BDD powder and Vylon [15]. In the current study, we have used both BDD/Nafion and BDD/Vylon inks for fabricating BDD film and needle electrodes. The electrodes have been characterized via cyclic voltammetry and attached to a plant to assess their potential for long-term use.

## 2. Experimental Section

BDD powder (BDDP) was prepared by depositing BDD onto the surface of natural diamond powder (particle diameter < 500 nm). Detailed experimental conditions have been reported in the literature [14,15].

**Figure 1:**
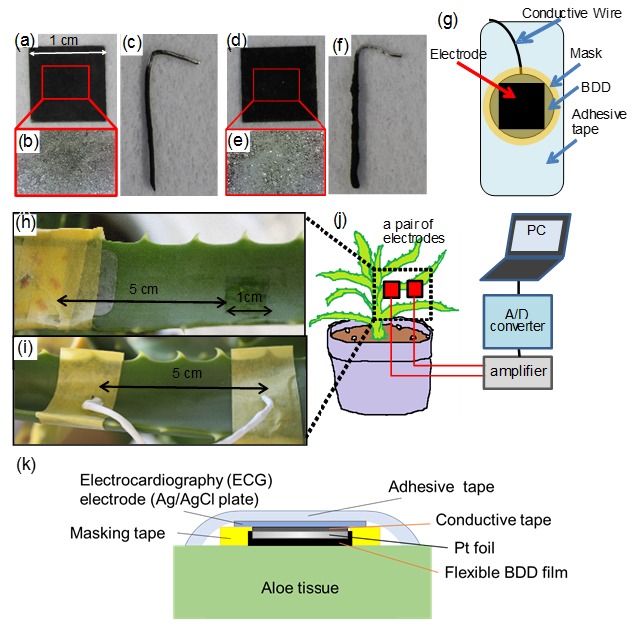
Images of the fabricated electrodes: (**a**) BDD/Nafion film, (**b**) surface of BDD/Nafion film, (**c**) BDD/Nafion needle, (**d**) BDD/Vylon film, (**e**) surface of BDD/Vylon film, surface of BDD/Vylon film, (**f**) BDD/Vylon needle. The structure of the sensor element for the BDD plate- or film-type electrodes is shown in (**g**). Images showing the attachment of the (**h**) BDD plate- or film-type sensor elements and (**i**) needle-type electrodes to the plant. (**j**) Schematic of the measurement system for plant monitoring. (**k**) Schematic cross-section of a film-type electrode installed on an *Aloe* leaf.

When preparing the BDD/Nafion electrodes, 2-propanol was added to an ion-exchange polymer dispersion (20 wt % Nafion (R), Sigma-Aldrich Japan Co., Ltd., Tokyo, Japan ) to achieve a dispersion with a 2-propanol/20 wt % Nafion (R) ratio of 1:1. BDDP and Nafion resins of equal weight were used to prepare the BDD/Nafion ink. The BDD/Nafion ink was then subjected to ultrasonic dispersion treatment for 5 min. Pt foils (10 mm × 10 mm, Nilaco Co., Ltd., Tokyo, Japan) were painted with the BDD/Nafion ink and heated in an oven at 60 °C for 1 h and then at 120 °C for 10 min to form flexible BDD electrodes (BDD/Nafion film, Figure 1a,b). Additionally, needle-type sensor elements (BDD/Nafion needle, Figure 1c) were also fabricated with Pt wire (diameter: 0.5 mm, painted length: 1.5 mm) through the same painting and heating process as above.

Next, for the preparation of the BDD/Vylon electrodes, BDDP, Vylon (amorphous co-polyester, VYLON®, TOYOBO Co., Ltd., Osaka, Japan), KE-1830 (Silicon resin, Shin-Etsu Chemical Co., Ltd., Tokyo, Japan), and toluene were taken in the weight ratio of 5:4:1:100. Vylon was cut and dissolved in toluene and KE-1830, and BDDP was then added to the resin mixture. The prepared BDD/Vylon ink was then painted on Pt foils and heated under conditions similar to those used in the preparation of the BDD/Nafion electrodes (Figure 1).

Polycrystalline BDD plate electrodes (10 mm × 10 mm, Element Six Ltd., Tokyo, Japan) were used as sensor elements. Each BDD plate and film-type electrode was attached with conductive carbon tape (Cat. No. 7311, Nisshin EM Co., Ltd., Tokyo, Japan) onto 3M™ Red Dot™ electrocardiogram (ECG) monitoring electrodes (Ag/AgCl) along with a pre-attached 2269TP lead wire to form the sensor electrode.

The measurement system setup and the sensor element structure are shown in Figure 1g, k, respectively, and have been previously described in the literature [7,8]. In this study, masking tape was added onto an ECG monitoring electrode around a BDD electrode, such that only the BDD electrode surface was in contact with the plant tissue. Each pair of electrodes was attached to the leaves of a potted *Aloe* with 15 leaves, 5 of which were utilized. After attaching the electrodes to the leaves, we covered them with masking tape to protect against rain and electromagnetic noise. Bioelectric potential changes in the plant were detected using the pairs of sensor electrodes and the signal was amplified using a handmade amplifier. Picolog (AC/DC Converter, ADC-24, Pico Technology Ltd., St Neots, UK) was set up to record the amplified potential gap data between each pair of electrodes every second.

The electrochemical properties of BDD electrodes were characterized by cyclic voltammetry (vs. Ag/AgCl, in 0.5 M H_2_SO_4_) with an electrochemical analyzer (model: 1200B, ALS. Co., Ltd., Tokyo, Japan) at a scan rate of 0.1 V/s and sampling interval of 0.001 V. A potential range from −0.5 V to 2.0 V was scanned, with measurement beginning at −0.2 V.

## 3. Results and Discussion

**Figure 2:**
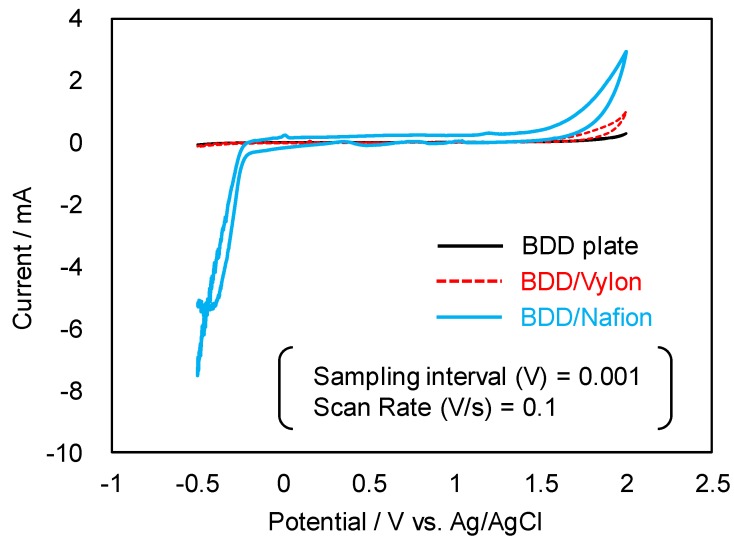
Cyclic voltammograms (vs. Ag/AgCl) of boron-doped diamond (BDD) electrodes in 0.5 M H_2_SO_4_.

Figure 2 shows the cyclic voltammograms (CV) (vs. Ag/AgCl) of BDD electrodes measured in 0.5 M H_2_SO_4_ solution. The background current of the BDD/Vylon film electrode was found to be low, and the CV of the film electrode was very similar to that of the BDD plate. Although the potential window of the BDD/Nafion film was slightly wider than that of the other electrodes, and the potential changes were less than or equal to −0.2 V, there were no obvious peaks in the potential range of −0.2 to 1.5 V. Therefore, the electrochemical properties of the three electrodes in the sensor element appeared to be suitable for plant monitoring.

**Figure 3:**
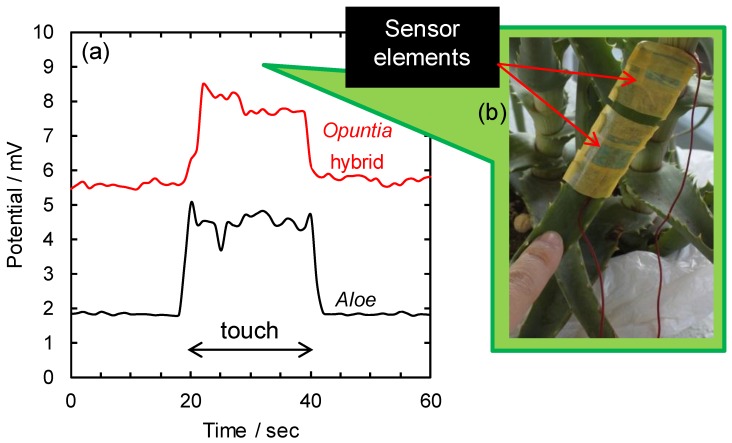
(**a**) Potential changes between a pair of BDD plate electrodes on potted *Aloe* and *Opuntia* hybrid for a finger touch lasting 20 s. (**b**) Image showing finger touch on a leaf of the potted *Aloe.*

As shown in Figure 3, touching a potted *Aloe* and potted *Opuntia* hybrid with a bare finger induced drastic potential changes. This is similar to the results in our previous report on potted *Opuntia* hybrid [7]. On the other hand, a finger touch with a latex examination glove either did not evoke any potential changes, or induced smaller potential changes compared to the changes observed when touched without a glove. The human body consists of cells with membranes, which have an associated membrane potential that is dependent on the distribution of various ionic species. A finger touch may act as an electrical stimulus for the plant. According to the literature on plant electrophysiology [4,5,6], electrical impulses may arise spontaneously, or result from stimulation. Once initiated, they can propagate to adjacent excitable cells. Changes in the transmembrane potential create a wave of depolarization, or action potential, which affects the adjoining and resting membranes. A membrane acquires its properties from its lipids and proteins, such as ion channels and transporters. Further, an electrical potential difference exists between the interior and exterior of the cells. As a charged object with mass moves between different gravitational potential points, biopotential changes are evoked [2,16,17]. Thus, a finger touch stimulus may result in potential changes. The induced potential changes between the BDD plate electrodes on the *Aloe* were almost the same from the points of view of response speed (less than one second) and mean order (few mV) as the changes seen for *Opuntia* hybrid. This result confirms that the system setup worked just as well on *Aloe* as it did in our previous study on *Opuntia* hybrid.

**Figure 4:**
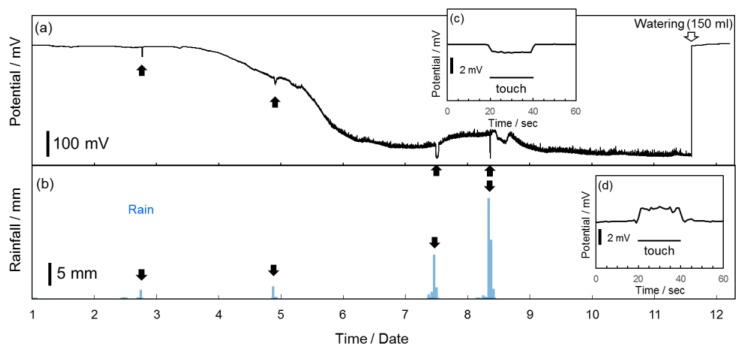
(**a**) Potential changes between a pair of BDD plate electrodes on *Aloe* during watering and rains over 11 days (from 2 to 13 October 2016). (**b**) Rainfall over the same 11 days. (**c**) Response to a finger touch stimulus when the plants were relatively water-starved. (**d**) Response after 150 mL watering.

Figure 4a shows the potential changes between a pair of BDD plate electrodes on the potted *Aloe* over a period of 11 days or more. Figure 4b is the rainfall log for the same period. Several potential changes appeared when there was rain, as pointed out by the black arrows, suggesting that the rainfalls caused the potential changes. Further, a swift and drastic increase in potential occurred upon watering on Day 11 after the potential slowly declined from Day 3 to Day 5, as shown in Figure 4a. This implies that while small but insufficient amounts of water (rains in Figure 4b) can change the biopotential of the plants immediately, the biopotential will not recover completely. As a result, the potential continued to decrease despite small amounts of rain. However, adding a sufficient amount of water (150 mL) allowed complete recovery of the biopotential on Day 11. The results showing the drastic move of the potential gap between electrodes and long-term effect of water corresponds to responses in electrical potential differences and in action potential on fruit trees described in [6], which were observed immediately after irrigation and was followed by a slow decrease. Next, the stability and reproducibility of the potential change response to finger touch on the *Aloe* plant was also assessed. The potential shown in Figure 4c increased immediately upon a finger touch stimulus; although, after watering, the potential decreased upon another finger touch stimulus, as shown in Figure 4d. These results suggest that the plant response is affected by their status and environment. Therefore, all the five types of BDD electrodes were set up on the potted *Aloe* to assess the response stability for long-term use. To maintain constant conditions for the potted *Aloe*, it was watered and moved into a room in which the temperature was maintained between 19 and 23 °C for at least 30 min before any finger touch stimulus was given.

**Figure 5:**
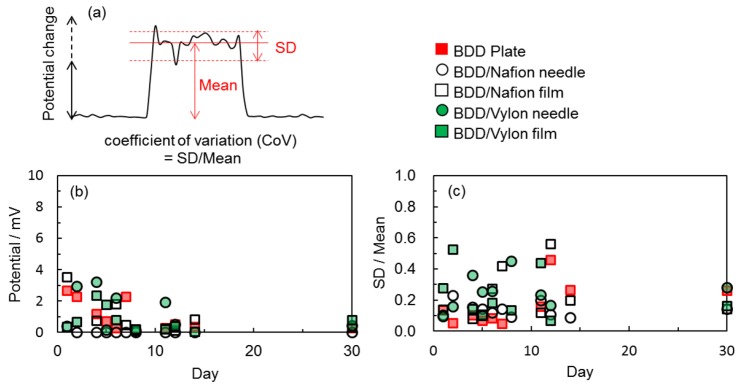
(**a**) Statistical analysis of the bioelectric potential changes upon a finger touch. (**b**) Mean (absolute value) of potential changes evoked by a finger touch on potted *Aloe* and (**c**) SD/mean (coefficient of variability) for the potential changes measured with each electrode.

Statistical analyses of the results were performed as follows. The coefficient of variability (CoV) [7] for the signal measured by each electrode was calculated using the mean and standard deviation (SD) of the bioelectric potential change (Figure 5a). The calculated means (absolute value) of potential changes between each pair of electrodes on the *Aloe* for a finger touch stimulus are plotted on Figure 5b. The CoV (mean/SD) values are also shown in Figure 5c.

To assess the reproducibility of the response, the response to finger touch stimulus was recorded at least 3 times for each pair of electrodes each day. For the first few days after setting up the electrodes on the *Aloe*, the means of the potential changes were spread out, as shown in Figure 5b. Although the mean values exhibited variability, the small CoV values in Figure 5c clearly imply that potential changes occurred. However, during the week following the first few days, the means became smaller. After the first two weeks, the means were completely stable and almost the same as the response on day 30. This trend was independent of the form of the electrode (plate/film or needle) or the electrode material (plate BDD, BDDP/Nafion, or BDDP/Vylon). Nafion is a sulfonated tetrafluoroethylene based fluoropolymer-copolymer, and is strongly acidic. Although a proton reduction peak was detected in the CV of BDDP/Nafion (as shown by the blue line in Figure 2), this did not seem to affect the trend. To estimate how the features of Nafion affected the signals between the electrodes on *Aloe*, at first an experiment to obtain a CV using BDD plate electrode attached to the potted *Aloe* was performed. The results showed that it was impossible to obtain any meaningful current due to the high resistance of the *Aloe*, suggesting that, in this case, signals that show potential changes between the electrodes are obtained not so much from ions carrying electric charge between the electrodes or reactions of ions on the electrode surfaces as they are from changes in the potential gap, caused by distribution of ions inside and outside of cells in a living body [4,6], between the electrodes. The result explains why the properties of Nafion did not affect the trend. In addition, it may be noted that the form of the BDD electrode (needle or plate/film type electrode) on the *Aloe* plant did not make an obvious difference in terms of the response to finger touch stimulus, and the materials (plate or powder-resin) used in the preparation of the electrodes did not significantly affect the results. Although the results of the study still need to be investigated further, it is reasonable to conclude that the inexpensive BDDP/resin electrodes used in this study were able to detect bioelectrical changes in living plant tissues as effectively as the BDD plate electrodes and can serve as a monitoring system for plants.

## 4. Conclusions

In this study, a plant monitoring system based on the detection of bioelectric potential with low-cost BDD electrodes consisting of BDD powder and resin was investigated. The results of the study allow us to conclude that BDD electrodes can be used to detect biopotential changes on a potted *Aloe* as changes in the potential gap between a pair of electrodes, regardless of the materials or forms of the electrode. The inexpensive and flexible BDD electrodes prepared from BDD powder and resin (Nafion or Vylon) performed just as reliably as the BDD plate electrodes. In addition, we conclude that BDD electrodes placed on live plant tissues can detect bioelectrical potential changes consistently and effectively. However, considering the cost of the materials, since generally Vylon is less expensive than Nafion, BDDP/Vylon would be favorable for practical use. Further, if it is wet or in a high-humidity atmosphere, the features of Nafion, which is strongly acidic and offers proton transportation mobility, might affect the response and the property as sensor. Then, we conclude that BDDP/Vylon would be best suited to monitoring biopotential changes in plants, while a form of sensor electrodes should be designed to fit the plants to which you would like to apply them. The reproducibility of the response of the BDD electrodes on the *Aloe* was also stable for at least 30 days. Thus, the results of the study prove the possibility of using inexpensive and flexible BDD electrodes for monitoring the bioelectric potential of live plants.

## Supplementary Materials

The following are available online at http://www.mdpi.com/1424-8220/17/7/1638/s1, Figure S1: Potential changes between a pair of Pt, Ag/AgCl and BDD plate electrodes on potted Opuntia hybrid for a finger touch lasting 20 s, Figure S2: (a) Potential changes between a pair of BDD plate electrodes on *Aloe* during watering and rains over 11 days (from June 1 to June 12, 2017). (b) Rainfall over the same 11 days.
