# Supplementary material for: Flexible Boron-Doped Diamond (BDD) Electrodes for Plant Monitoring
Source: Sensors (Basel). 2017 Jul 15;17(7):1638. doi: 10.3390/s17071638 (PMC5539713; doi:10.3390/s17071638)
Supplement: Supplementary file 1 [file sensors-17-01638-s001.pdf]

Supplementary data

Flexible Boron-Doped Diamond (BDD) Electrodes for Plant Monitoring

Shoko Tago, Tsuyoshi Ochiai, Seitaro Suzuki, Mio Hayashi,

Takeshi Kondo, and Akira Fujishima

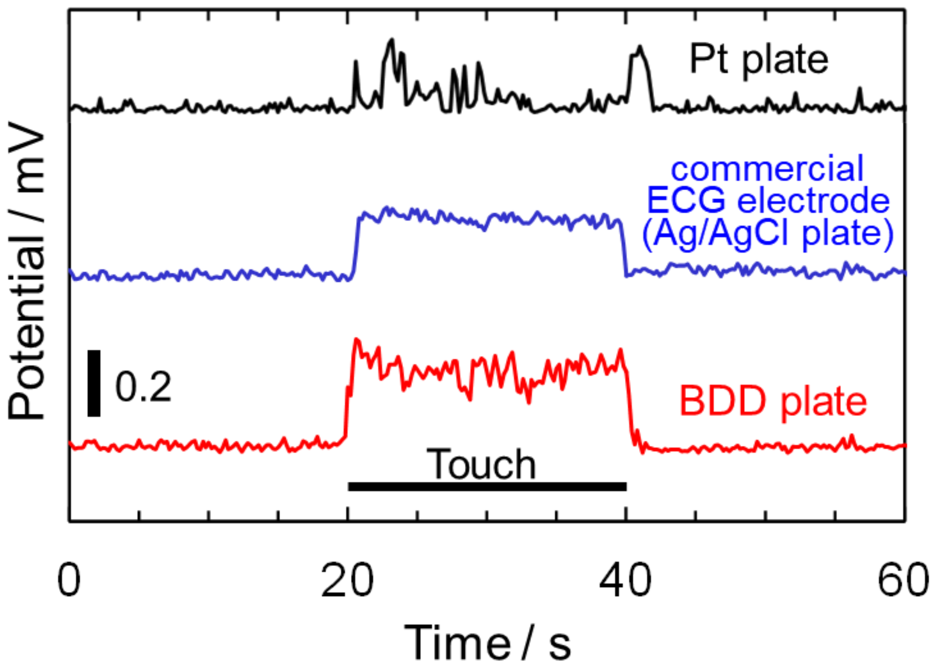

Figure S1 Potential changes between a pair of Pt, Ag/AgCl and BDD plate electrodes on potted *Opuntia* hybrid for a finger touch lasting 20 s.

Reproduced with permission from *ECS Trans.*, 75, 233 (2016). Copyright 2016 ECS - The Electrochemical Society.

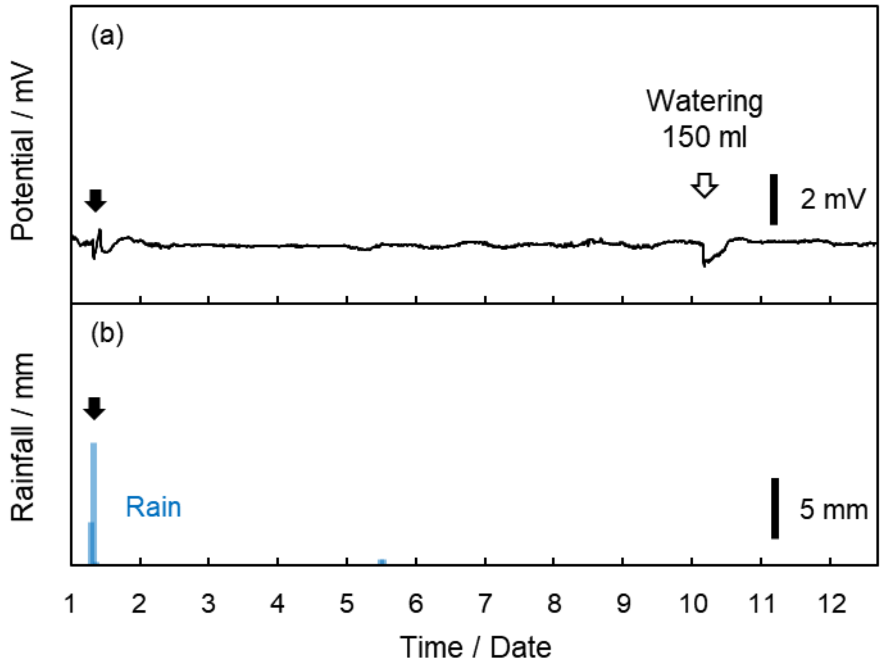

Figure S2 (a) Potential changes between a pair of BDD plate electrodes on *Aloe* during watering and rains over 11 days (from June 1 to June 12, 2017). (b) Rainfall over the same 11 days.
